# Beneficial effects of xenon inhalation on behavioral changes in a valproic acid-induced model of autism in rats

**DOI:** 10.1186/s12967-019-02161-6

**Published:** 2019-12-03

**Authors:** A. P. Dobrovolsky, V. R. Gedzun, V. I. Bogin, D. Ma, T. E. Ichim, Iu. A. Sukhanova, A. V. Malyshev, V. A. Dubynin

**Affiliations:** 1 Pirogov Russian National Research Medical University, Ostrovitianov str. 1, Moscow, 117997 Russia; 2 Department of Biology, Lomonosov Moscow State University, Moscow, Russia; 3 Nobilis Therapeutics Inc, Portland, OR USA; 4 Anaesthetics, Pain Medicine & Intensive Care, Department of Surgery & Cancer, Imperial College London, London, UK

**Keywords:** Xenon, Autism, ASD, NMDA, VPA model, Behavioral testing

## Abstract

**Background:**

Xenon (Xe) is a noble gas that has been used for the last several decades as an anesthetic during surgery. Its antagonistic effect on glutamate subtype of NMDA (*N*-methyl-d-aspartate) receptors resulted in evaluation of this gas for treatment of CNS pathologies, including psychoemotional disorders. The aim of this study was to assess the behavioral effects of acute inhalation of subanesthetic concentrations of Xe and to study the outcomes of Xe exposure in valproic acid (VPA)-induced rodent model of autism.

**Methods:**

We have conducted two series of experiments with a battery of behavioral tests aimed to evaluate locomotion, anxiety- and depression-like behavior, and social behavior in healthy, VPA-treated and Xe-exposed young rats.

**Results:**

We have shown that in healthy animals Xe exposure resulted in acute and delayed decrease of exploratory motivation, partial decrease in risk-taking and depressive-like behavior as well as improved sensorimotor integration during the negative geotaxis test. Acute inhalations of Xe in VPA-exposed animals led to improvement in social behavior, decrease in exploratory motivation, and normalization of behavior in forced-swim test.

**Conclusion:**

Behavioral modulatory effects of Xe are probably related to its generalized action on excitatory/inhibitory balance within the CNS. Our data suggest that subanesthetic short-term exposures to Xe have beneficial effect on several behavioral modalities and deserves further investigation.

## Background

Autism Spectrum Disorder (ASD) is a neurodevelopmental disorder characterized by impaired communication and social interactions combined with restricted repetitive and stereotyped behaviors, activities and interests [1–3]. According to most recent CDC report the overall prevalence of ASD in the US was 16.8 per 1000 (one in 59) children aged 8 years (https://www.cdc.gov/ncbddd/autism/data.html). Autism is known as a “spectrum” disorder because there is wide variation in the type and severity of symptoms from one clinical case to another [3]. Etiology of ASD appears to be rather heterogeneous and includes both genetic predisposition and environmental exposure at the early stages of development [4]. There are a number of probable mechanisms underlying the development of ASD, including alterations in glutamatergic/GABAergic neurotransmission, inflammatory signals, and oxidative stress-related systems, which also may explain neurobiological susceptibilities to adverse environmental exposures [5]. Such heterogeneity suggests that no single treatment or diagnostic biomarker is likely to be found for autism [3].

The most frequently observed hyper-responsiveness and increased anxiety in ASD are largely explained as the imbalance of inhibitory and excitatory processes in the brain, mainly in limbic structures [6]. Increasing evidence indicates that dysfunction of NMDA (*N*-methyl-d-aspartate) receptors (NMDARs) at excitatory synapses is associated with ASD [7].

Animal models that recapitulate glutamatergic neurotransmission deficits—with corresponding autism-like behavioral readouts—are useful in evaluating novel therapies that could be translated to the treatment of autism [8]. Valproic acid (VPA)-induced rodent model of autism is a well-studied and robust model with strong predictive validity [9]. While transgenic models have become more popular, they reflect single gene mutations where it is generally agreed that the most prevalent “idiopathic” ASD is a complex genetic disorder where environmental/epigenetic origins play a significant role. VPA exposure is a risk factor for developing autism [10] and VPA-exposed animals exhibit autism-like behaviors including deficits in sensorimotor gating, stereotyped movements and abnormal social behaviors [9].

Currently, there is no cure available for autism [9]. Therapeutic options to improve clinical deficits in ASD include pharmacological interventions [11, 12], cognitive behavioral therapy [13, 14], and experimental approaches, such as the use of noninvasive transcranial brain stimulation [15]. Children with ASD are more sensitive to pharmacological interventions and more likely to have adverse effects than children without ASD. There clearly exists a tremendous unmet need in finding new safe and efficacious approaches to treatment of ASD.

Xenon (Xe) is a noble gas which has been used for the last several decades as an anesthetic during surgery. In comparison with other gaseous anesthetics it has beneficial properties such as rapid introduction into the brain, hemodynamic stability profile with little or no toxicity, and its lack of metabolizm [16]. A recent clinical trial has shown that inhalation of 30% Xe within a delayed time frame after birth asphyxia is apparently safe for newborns [17]. Further preclinical models suggest potential clinical benefit of subanesthetic doses of Xe in indications such as traumatic brain injury, ischemic or hemorrhagic stroke, perinatal hypoxic-ischemic brain injury, coronary artery bypass graft surgery, organ protection during transplantation, chronic pain, addiction and post-traumatic stress disorder [18–25]. Since the discovery of Xe’s inhibitory effect on NMDAR channels [26] extensive studies in this field have revealed more of the underlying mechanisms which include reduction of excitatory neurotransmission through downregulation of 5-HT_3_ [27], nicotinic acetylcholine and AMPA receptors [28, 29] as well as potassium KATP [30], and HCN channels [31].

Xe-oxygen mixtures have been successfully employed in a recent open-label study to treat panic disorder (PD) by Dobrovolsky and colleagues. Six sessions of 4-min Xe inhalations have shown potency to reduce the severity of panic attacks and the severity of depressive disorders in patients with PD. The authors have proposed that Xe should be further studied as an alternative to benzodiazepines as a safe modality in the treatment of anxiety disorders [32]. With regard to ASD, the ability of Xe to reduce excitatory neurotransmission (E) and increase in inhibitory neurotransmission (I) suggests it may restore a putative imbalance in E/I present in patients with ASD. To date, there is no literature available on the behavioral effects of Xe administration in rodent models of autism.

The aim of this work was to assess the behavioral effects of acute inhalation of subanesthetic doses of Xe and to study the outcomes of Xe exposure in VPA-induced rodent model of autism. We have conducted two series of experiments with a battery of behavioral tests aimed to evaluate locomotion, anxiety- and depression-like behavior, and social behavior in normal, VPA-treated and Xe-exposed young rats.

## Materials and methods

### Animals

Wistar rats (N = 96) of both sexes from 10 litters were used in the study. The day of birth was counted as postnatal day (pnd) 0. Animals were housed in a 12-h dark/light cycle in a temperature-controlled environment with food and water ad libitum. All efforts were made to minimize the number of animals used. All protocols and procedures were conducted in accordance with Moscow State University’s Committee on Biomedical Research Ethics.

### Treatment

In the current study we have conducted two series of experiments (series 1 and 2). In series 1 we assessed the effects of acute subanesthetic doses of Xe on normal rats in a battery of behavioral tests. After the successful completion of series 1, we assessed the effect of Xe inhalations on behavior in VPA-treated rats in series 2.

In series 1, rat pups (pnd 20) were first acclimated to the gas exposure apparatus for 10 min. One day later (pnd 21) rats from each litter were randomly assigned to “Xe” (N = 23) or “Control” (N = 21) groups and were treated with 25% ± 2.5% Xe or atmospheric air for 10 min respectively. Following exposure, rats were removed from the apparatus and placed in individual cages for 10 min followed by behavioral testing. Xe/air exposures were conducted before each test in the battery (except for elevated O-maze). A total of three exposure sessions were carried out in series 1 (Table 1).

**Table 1:** Timeline of experimental procedures in series 1 and 2

pnd	Series I	Series II
0	Day of birth	
6–11	No treatment	VPA (150 mg/kg)
12		VPA (150 mg/kg), adaptation in Xe/air-exposure chamber
13		Xe/air inhalation followed by negative geotaxis test
17		Xe/air inhalation followed by gait test
20	Acclimation to Xe/air-exposure chamber	
21	Xe/air inhalation followed by open field test	
25	Xe/air inhalation followed by elevated plus maze	
28		Xe/air inhalation followed by test for social novelty
32		Xe/air inhalation followed by social play behavior test
35	Xe/air inhalation followed by forced swim test	
40	Elevated O-maze	

In series 2 rat pups (pnd 6) from each litter were treated with either saline or valproic acid injections (Sigma Aldrich, USA) dosed at 150 mg/kg intraperitoneally (“Control” and “VPA” group respectively) once each day for 1 week (pnds 6–12). VPA-induced model of ASD was selected as it is the most common model of induction of autism-like behaviors in rodents [33, 34]. On pnd 12 rat pups were acclimated to the gas exposure apparatus for 10 min. On pnd 13 “VPA” and “Control” groups were divided into “VPA-Xe” (N = 14), “VPA-air” (N = 14), “Cont-Xe” (N = 11), “Cont-air” (N = 13) groups of animals. Rats were treated with 25% ± 2.5% Xe or atmospheric air for 10 min respectively. Following exposure, rats were removed from the apparatus and placed in individual cages for 10 min followed by behavioral testing. Xe or air exposures were conducted before each task in the battery; a total of seven inhalation sessions were carried out in series 2, with 3–4 days between the inhalation + testing (Table 1).

### Xenon exposure apparatus

**Fig. 1:**
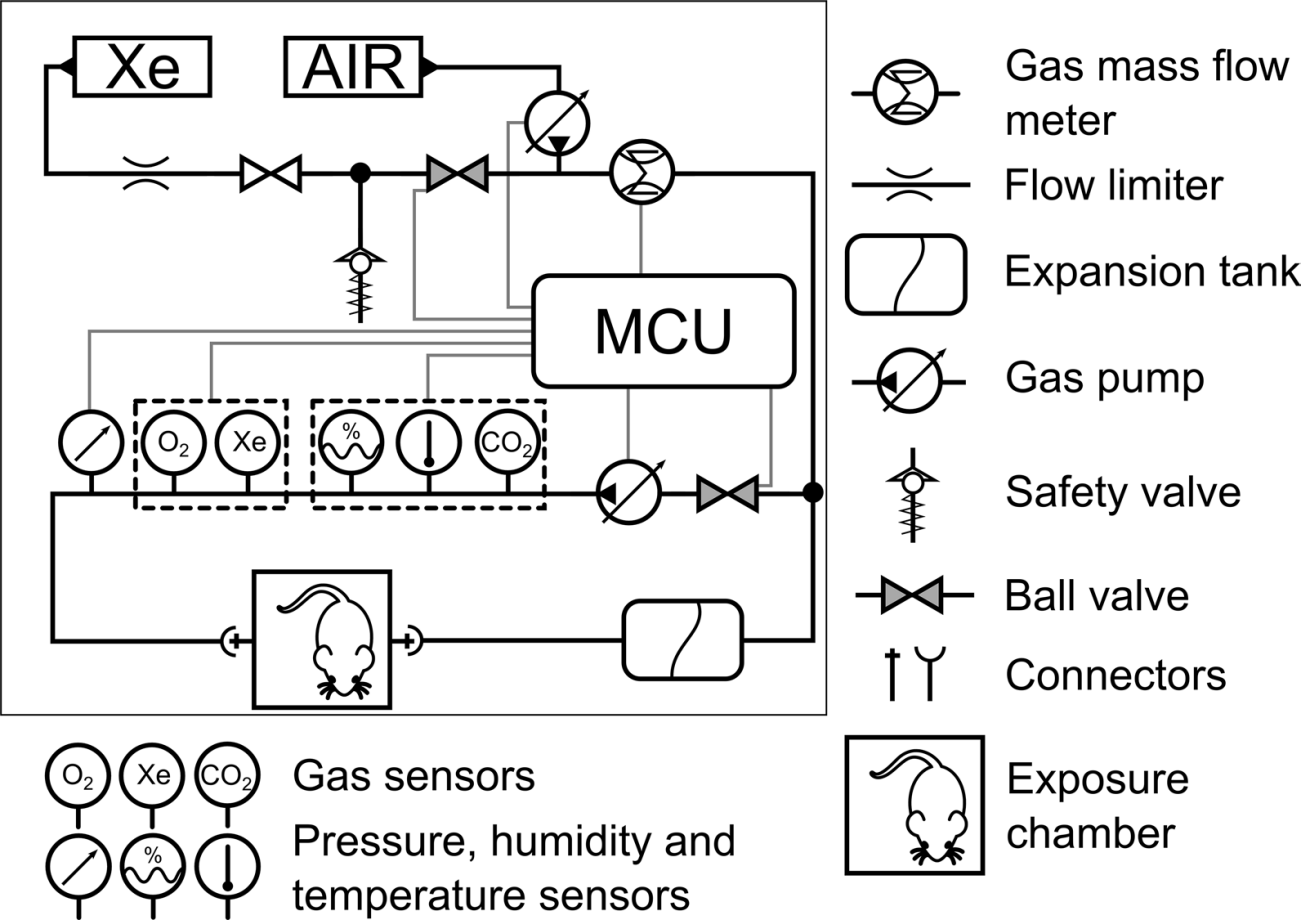
Schematic representation of xenon exposure apparatus. *MCU* micro control unit

We used a custom system to expose animals to 25% ± 2.5% Xe gas (“Medksenon” ^®^, Russia). The apparatus consisted of 14 × 14 × 7 cm sealable Plexiglas chamber for exposure of three neonatal rats at a time (Fig. 1). The delivery (rate and concentration) both of Xe and atmospheric air (as needed to maintain 21% O_2_ concentration) was regulated by using flow meters (D6F-P0010A2, Omron, Japan). The animals were placed in a chamber and after that the gas mixture was introduced therein. We have started 10 min countdown when the Xe concentration in the chamber reached 25%. The required concentration was acquired within 30 s. Xe, oxygen, carbon dioxide, pressure, temperature and humidity were all monitored by sensors in the system and were adjusted as needed by the micro control unit (MCU) and supporting equipment to maintain set levels. The carbon dioxide level was kept less than 0.5% with soda lime (“Sodasorb” ^®^, GSP, USA). An identical system was used for air exposures except that only room air was supplied. Xe treatment was performed before each behavioral task (except for elevated O-maze). After 10 min rats were removed from the exposure apparatus and placed in individual cages until behavioral testing.

### Sensorimotor development assessment

Examinations of the effects of VPA on neurobehavioral development were carried out only in series 2. Testing started 1 day after the last VPA or vehicle administration and was preceded by Xe/air exposure. The assessment of sensorimotor development was carried out in accordance with the following schedule: negative geotaxis test (pnd 13), gait reflex (pnd 17). All motor development tests were adapted from Altman [35].

For the negative geotaxis test, pups were placed on a 30-cm incline plane (20°), in a head down position. The time required to reorient to a head up position was recorded; the cut-off time was 30 s. For the gait reflex, pups were placed in the center of a 10-cm diameter circle printed on white paper, and the time taken to move off the circle was recorded.

### Behavioral testing

#### Open field (OF)

To assess locomotion and responsiveness of the animals to a novel environment, we tested rats on pnd 21 in open field apparatus as previously described [36].

#### Elevated plus maze (EPM)

To measure anxiety-like behavior in rats we tested animals in the elevated plus maze (OpenScience Ltd, Russia) on pnd 25 as previously described [37]. The number of entries in open and closed arms as well as in the central part of the maze was counted for 5 min. Also, frequency of grooming, rearing, head dips and approaches to outer edge of the open arms were recorded during the experiment.

#### Social interaction behaviors

##### Test for social novelty

The apparatus for sociability and preference for social novelty test comprised a T-shaped opaque Plexiglas box with 30 cm high walls. The base of the “T” is 16 × 50 cm and serves as the start area while the lateral chambers are 10 × 50 cm (goal arms) and are used to hold a stimulus (Fig. 9b). The floor was divided into equal sections for assessment of locomotor activity. The dead-ends of the goal arms were separated with wire mesh to form a corner large enough to hold a single adult rat. Before the experiment an unfamiliar adult female rat was placed in the right chamber and tested animal’s dam was placed in the left goal arm of the maze. The subject rat on pnd 28 was placed back to the wall in the base arm and allowed to explore the goal arms. The following parameters were monitored and recorded for 5 min: duration and number of direct contacts between the subject rat and unfamiliar female/dam, and the latency to leave the starting area. The rat is considered to be in the chamber when its head and four paws have entered into the chamber.

##### Social play behavior

The test was performed under dim white light and familiar conditions (in the open field apparatus) on pnd 32. The test consisted of placing two animals from the same group but from different litters and cages into an open field. Pairs were tested in a randomized order for groups and the animals did not differ by more than 15 g in body weight. Behavior was monitored and registered with webcam for 3 min. Number of attacks (pinning and pouncing) and number of sniffings were counted.

##### Forced swim test (FS)

To assess any depression-like effects of VPA treatment, and potential antidepressant-like effect of Xe inhalation, rats were exposed to a forced swim test on pnd 35 in a transparent Plexiglas cylinder and the test was performed as previously described [37].

##### Elevated O-maze (OM)

In order to assess the delayed effects of Xe administration on anxiety-like and exploratory behavior of rats we performed the elevated O-maze on pnd 40. The apparatus (OpenScience Ltd, Russia) consists of two open (stressful) and two enclosed (safe) elevated arms that form a circle with an external diameter of 105 cm. The closed arms consist of 27-cm high walls and the width of both closed and open arms alleys of 10 cm. The open arms were illuminated with two bright lamps (60 W) located at a distance of 25–30 cm. The rats were placed in one of the enclosed arms and the latency to leave the closed arm and the number of the open arm entries as well as the number of head dips and the time spent in closed arms was measured for 5 min.

### Statistics

Statistical analysis was performed by using the Statistical Package STATISTICA 10 and GraphPad Prism 6. Data were assessed for normality using the Shapiro–Wilk’s W to determine whether to use parametric or non-parametric statistical tests. For a pair comparison we used either the Student’s T-test or the Mann–Whitney U-test and Fisher’s exact test for normally and abnormally distributed data, respectively. When comparing multiple groups, we used One-way ANOVA followed by Sidak’s multiple comparison test. Per-minute differences in performance in OF and FS tests among groups were evaluated by two-way ANOVA repeated measures with subsequent application of Dunnett’s or Sidak’s multiple comparison test. The normally distributed data are expressed as mean ± standard error of the mean (SEM), and non-normal data—as the box and whiskers plot. In the current study the data analysis has not shown any effects of Sex or Treatment X Sex interaction on analyzed parameters. The data are presented for the whole sample. Statistical differences were considered significant when a p value was less than 0.05.

## Results

### Series 1: healthy rats

#### Open field test

**Fig. 2:**
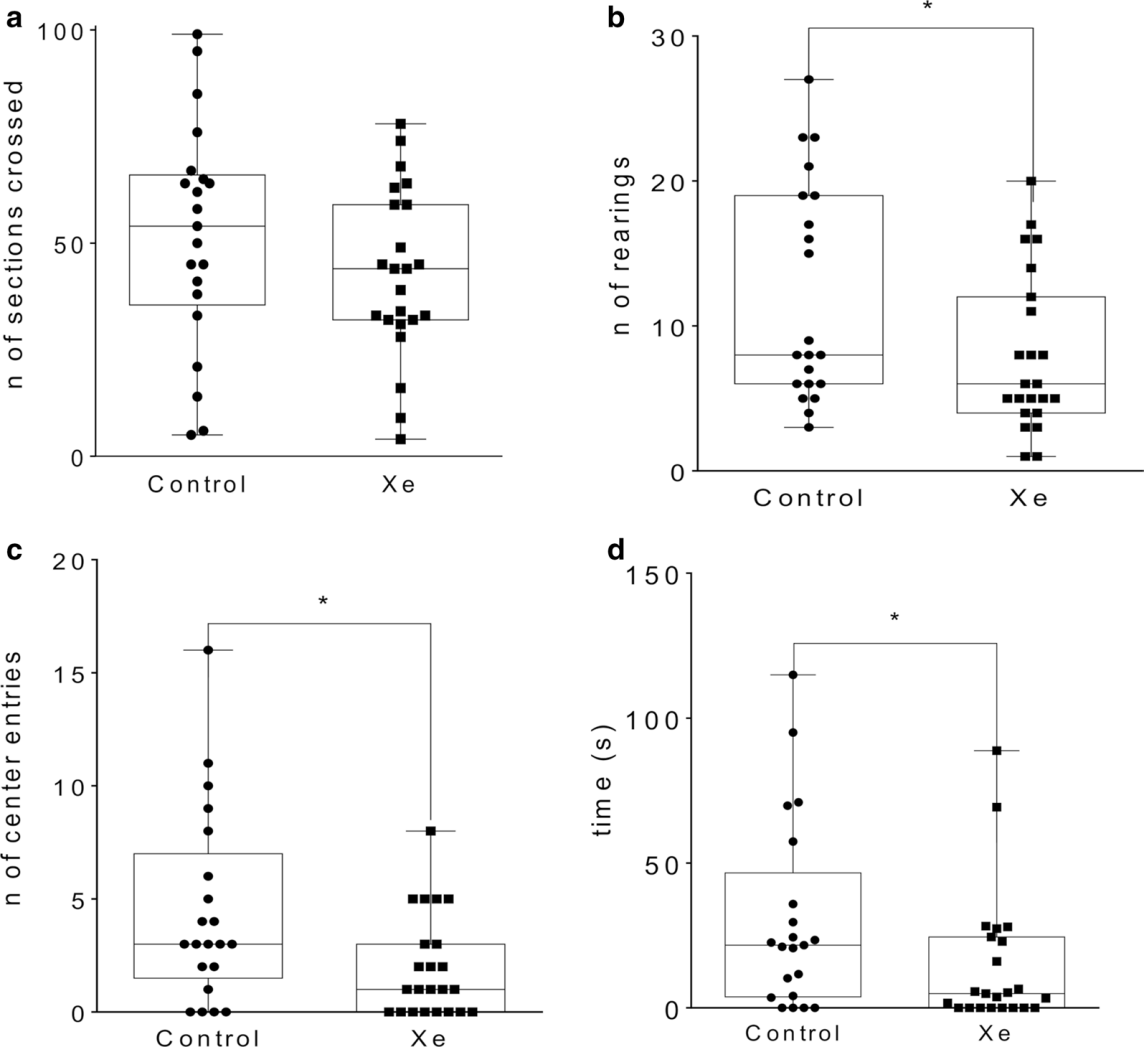
Effects of acute Xe inhalation on the behavior of healthy intact rats in open field test on pnd 21. Values represent number of sections crossed (**a**), rears (**b**) and center entries (**c**) as well as time spent (in seconds) in the center (**d**) within 5 min observation. *p <  0.05 represent significant differences vs. Control group (Mann–Whitney U-test), $—trend to significant difference 0.10 ≥ p ≥ 0.05. Data are presented as box and whiskers plot. N = 21–23 rats/treatment

A single Xe inhalation (25%, 10 min) prior to OF testing resulted in significant decrease of center entries (p = 0.021) and number of rears (p = 0.045) in Xe-exposed rats in comparison with air-exposed animals (Fig. 2). Also, there was a tendency towards the decrease in time spent in the center of the arena (p = 0.052) in “Xe” group vs. “Control” group. There was no difference in locomotor and grooming activity between groups.

#### Elevated-plus maze

**Fig. 3:**
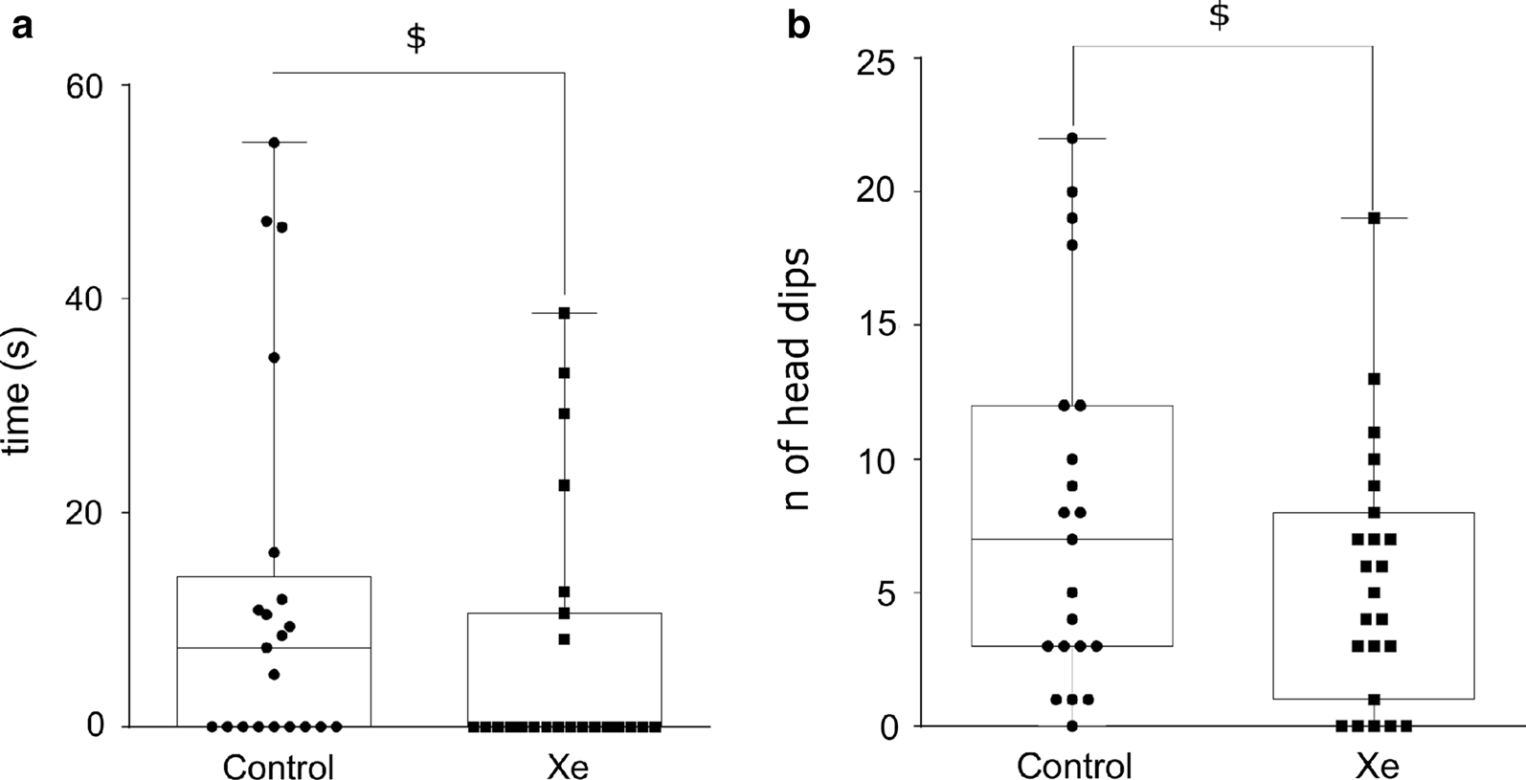
Effects of acute Xe inhalation on the behavior of normal rats in elevated plus maze on pnd 25. Time spent (in seconds) on the distal segments of open arms (**a**) and number of head dips (**b**) were measured within 5 min observation. Trends to significant difference—$—0.10 ≥ p ≥ 0.05 represent differences vs. Control group (Mann–Whitney U-test). Data are presented as box and whiskers plot. N = 21–23 rats/treatment

Xe-exposed and air-exposed rats showed no differences in time spent in the open arms, time spent in the closed arms, time spent in the center, number of open and closed arm entries, rears and grooms (p > 0.45; data not shown). However, Xe-exposed rats did show a tendency towards decreased time spent on the distal edge of the open arms (p = 0.10) and of the number of head-dips into open arms (p = 0.07) but these differences were not significant (Fig. 3).

#### Forced swim test

**Fig. 4:**
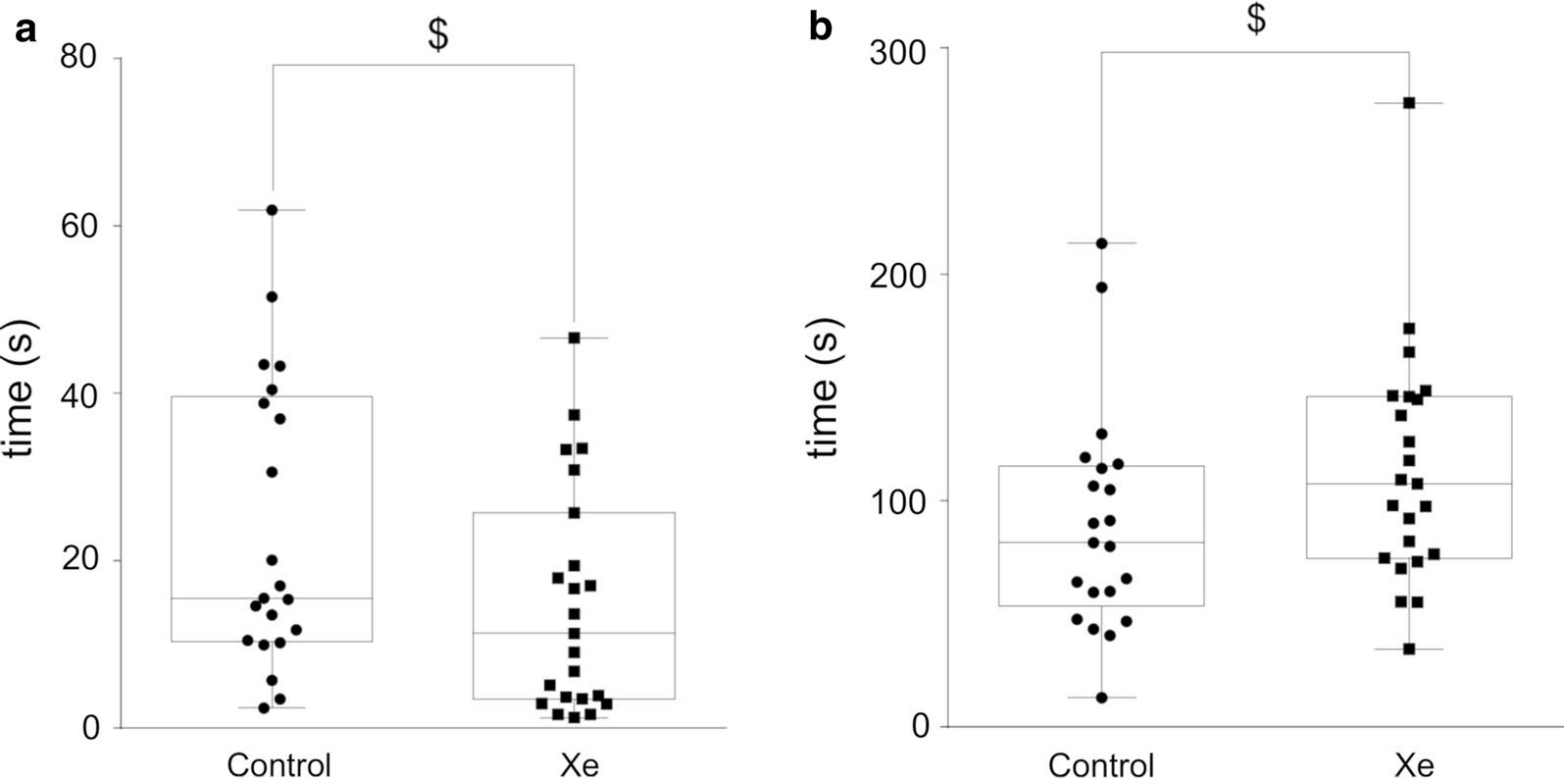
Performance of rats in forced swim test after acute Xe inhalation on pnd 35. Time spent immobile (in seconds) (**a**) and latency to first immobilization (in seconds) (**b**) were measured within 5 min observation. $—0.10 ≥ p ≥ 0.05 represent trend towards differences vs. Control group (Mann–Whitney U-test). Data are presented as box and whiskers plot. N = 21–23 rats/treatment

In the FST test Xe-exposed rats showed a trend towards decreased time spent immobile (p = 0.055) and increased latency to immobility (p = 0.067) in comparison with control group (Fig. 4). There were no differences in time spent climbing and time spent swimming between two groups (p > 0.05).

#### O-maze

**Fig. 5:**
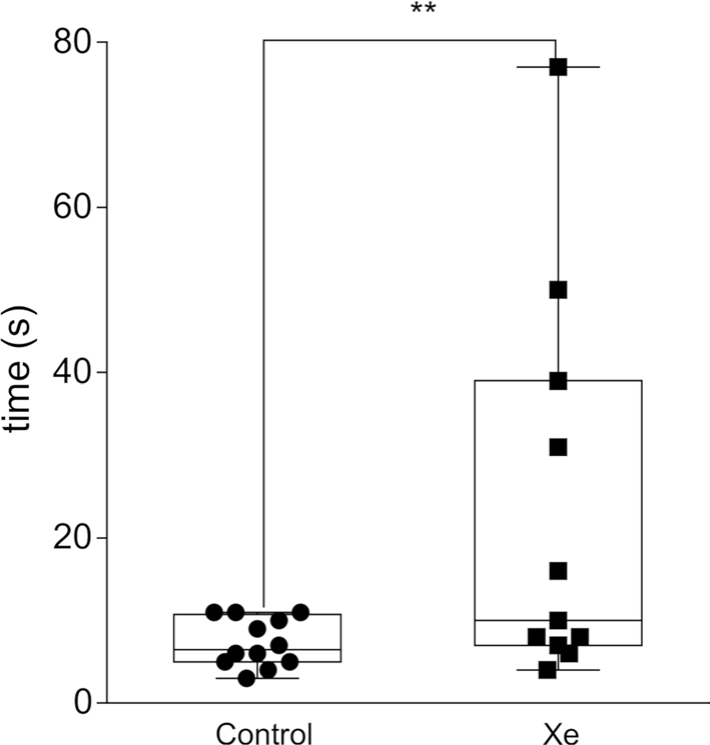
Delayed effects of previous Xe inhalations on the behavior of normal rats in elevated O-maze on pnd 40. Values represent latency (in seconds) to leave the closed arm of the maze. **p < 0.01 represent significant differences vs. Control group (Mann–Whitney U-test). Data are presented as box and whiskers plot. N = 21–23 rats/treatment

We have studied the behavior of rats in the elevated O-maze on pnd 40 (without pre-exposure to Xe). There was an increase in latency to leave the starting arm in Xe-exposed animals in comparison with control (p < 0.01) (Fig. 5). There were no changes in the time spent in the starting arm, the amount of open arms entries and the number of head dips between groups (p > 0.05).

### Series 2: VPA-exposed rats

#### Tests on sensorimotor development

##### Negative geotaxis test

**Fig. 6:**
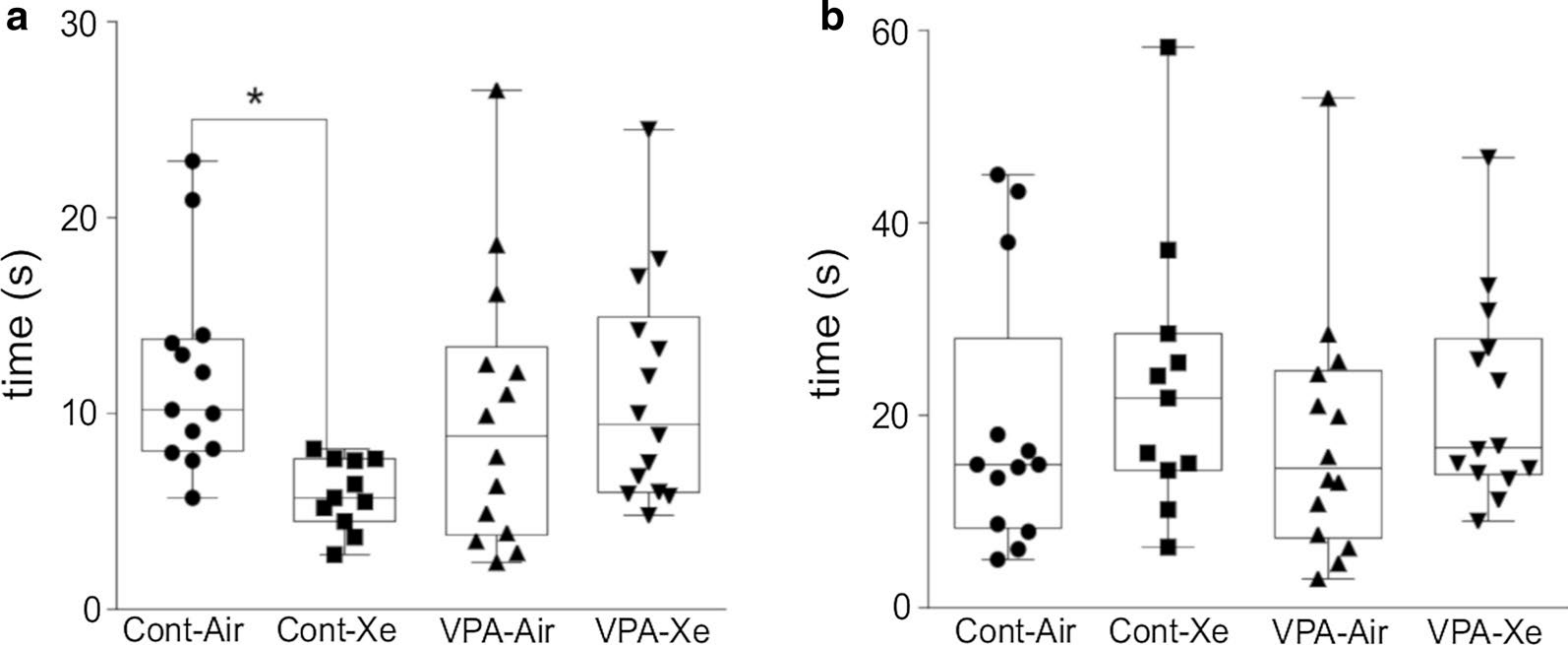
Effects of VPA treatment and acute Xe inhalation on the neurobehavioral development of rat pups. Values represent mean latency times (in seconds) to perform the following tests: negative geotaxis on pnd 13 (**a**), gait on pnd 17 (**b**). *p < 0.05 represent significant differences vs. Control group (Dunnett’s multiple comparisons test). Data are presented as box and whiskers plot. N = 11–14 rats/treatment

The results of one-way ANOVA revealed significant treatment effect (F_3, 46_ = 2.79; p = 0.05) in the negative geotaxis test on pnd 13. Post hoc analysis showed a significant effect of acute Xe inhalation as the “Xe” group of animals had increased latency to turn around in comparison with control animals (p = 0.02) (Fig. 6a). There were no differences between “VPA”, “VPA + Xe” and “Control” groups in this test.

##### Gait test

The results of one-way ANOVA revealed no significant treatment effect (F_3, 46_ = 0.48; p = 0.70) in the gait test on pnd 17 (Fig. 6b).

#### Open field test

**Fig. 7:**
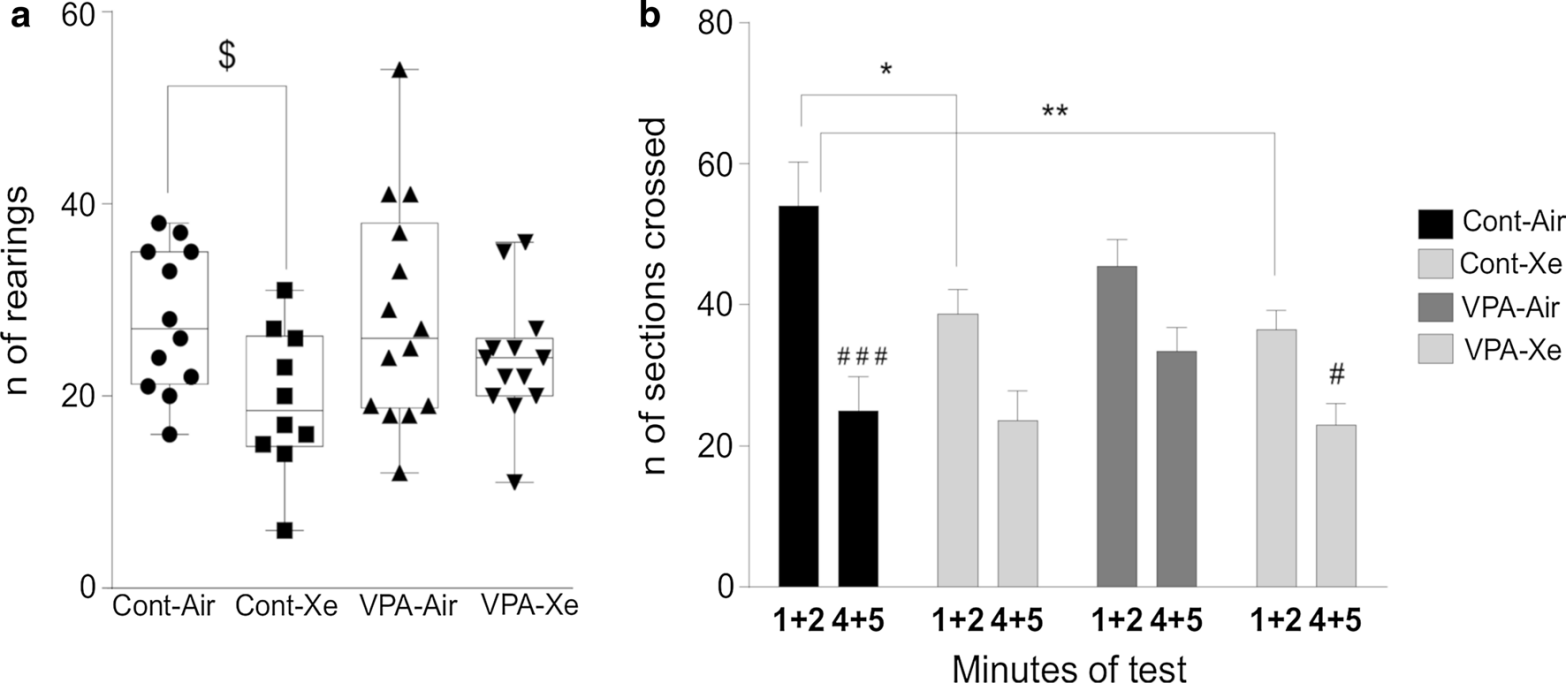
Effects of VPA treatment and acute Xe inhalation on the behavior of rats in open field test on pnd 21. Number of total rearings (**a**) and number of sections crossed within first 2 and last 2 min of observation (**b**) were measured within a 5-min observation. *p < 0.05, **p < 0.01 represent significant differences vs. Control group; ^#^p < 0.05, ^###^p < 0.001—significant differences within the group between first 2 and last 2 min of observation and $—0.10 ≥ p ≥ 0.05 represent a trend towards differences vs. Control group (Sidak’s multiple comparisons test) Data are presented as box and whiskers plot. N = 11–14 rats/treatment

The result of two-way repeated measures with ANOVA revealed significant treatment effect (F_3, 44_ = 2.874; p = 0.047) and Time (F_1, 44_ = 38.47; p < 0.0001) and a trend towards significance for interaction (F_3, 44_ = 2.14; p = 0.10) on locomotion in the OF test. *Post*-*hoc* analysis has shown a significant decrease in the number of crossings in “Xe” (p = 0.05) and “VPA-Xe” (p = 0.007) groups in comparison with “Cont” group of animals and didn’t differ between two Xe-exposed groups. Moreover, this decrease was observed only during the first 2 min of the experiment (Fig. 7b). There were no effects of VPA exposure on locomotion of rats in this test. There was also a trend towards significance for the treatment (F_3, 44_ = 2.55; p = 0.07) on the total number of rears with post hoc revealing a tendency towards decreased vertical activity in Xe-exposed rats in comparison with controls (p = 0.08) and no differences between other groups of animals (Fig. 7a). Moreover, one-way ANOVA did not reveal any significant effects of treatment on the number of center entries (F_3, 44_ = 0.86; p = 0.47) and grooming (F_3, 44_ = 0.66; p = 0.58).

#### Elevated-plus maze

**Fig. 8:**
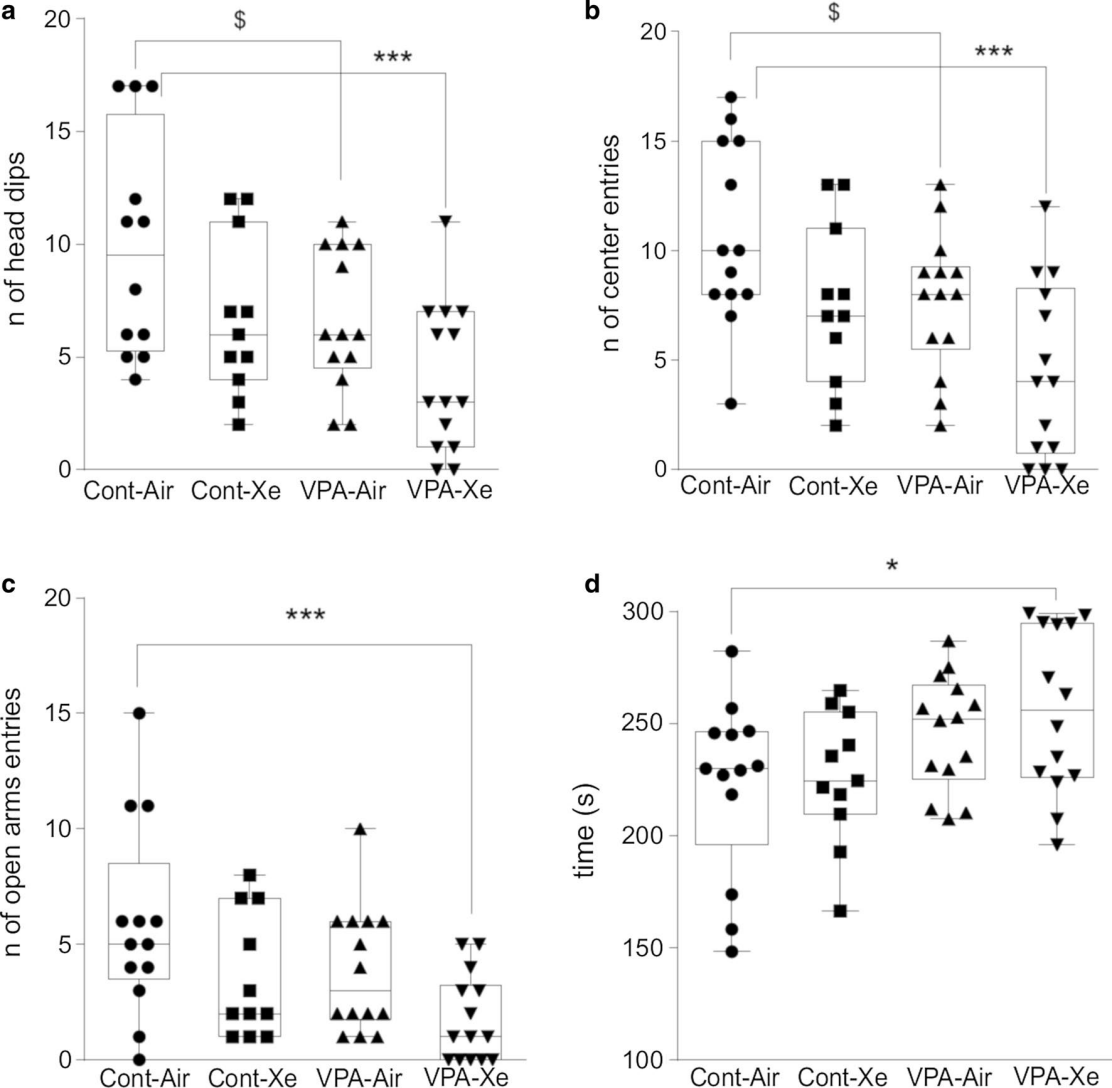
Effects of VPA treatment and acute Xe inhalation on the behavior of rats in elevated plus maze on pnd 25. Number of head dips (**a**), center entries (**b**), open arm entries (**c**) and time spent (in seconds) in the closed arms (D) were measured within 5 min observation. *p < 0.05 and ***p < 0.001 represent significant differences vs. Control group (Dunnett’s multiple comparisons test), and $—0.10 ≥ p ≥ 0.05 represent trend towards differences vs. Control group (Sidak’s multiple comparisons test). Data are presented as box and whiskers plot. N = 11–14 rats/treatment

The results of one-way ANOVA revealed a significant treatment effect on the number of head dips (F_3, 48_ = 5.172; p = 0.004), number of center entries (F_3, 48_ = 6.176; p = 0.001), number of open arm entries (F_3, 48_ = 4.361; p = 0.008) and time spent in the closed arms (F_3, 48_ = 2.998; p = 0.04). Post hoc analysis revealed a trend towards reduction of head dips (p = 0.09) and center entries (p = 0.10) in “VPA” group in comparison with “Cont” group. Also, there was a significant reduction in head dips (p < 0.001), center (< 0.001) and open arm (p = 0.002) entries as well as increased time spent in the closed arms of the maze (p = 0.033) in “VPA + Xe” group in comparison with “Cont” group (Fig. 8). Xe inhalations didn’t affect the behavior of healthy animals in this test.

#### Social behavior

##### Test for social novelty

**Fig. 9:**
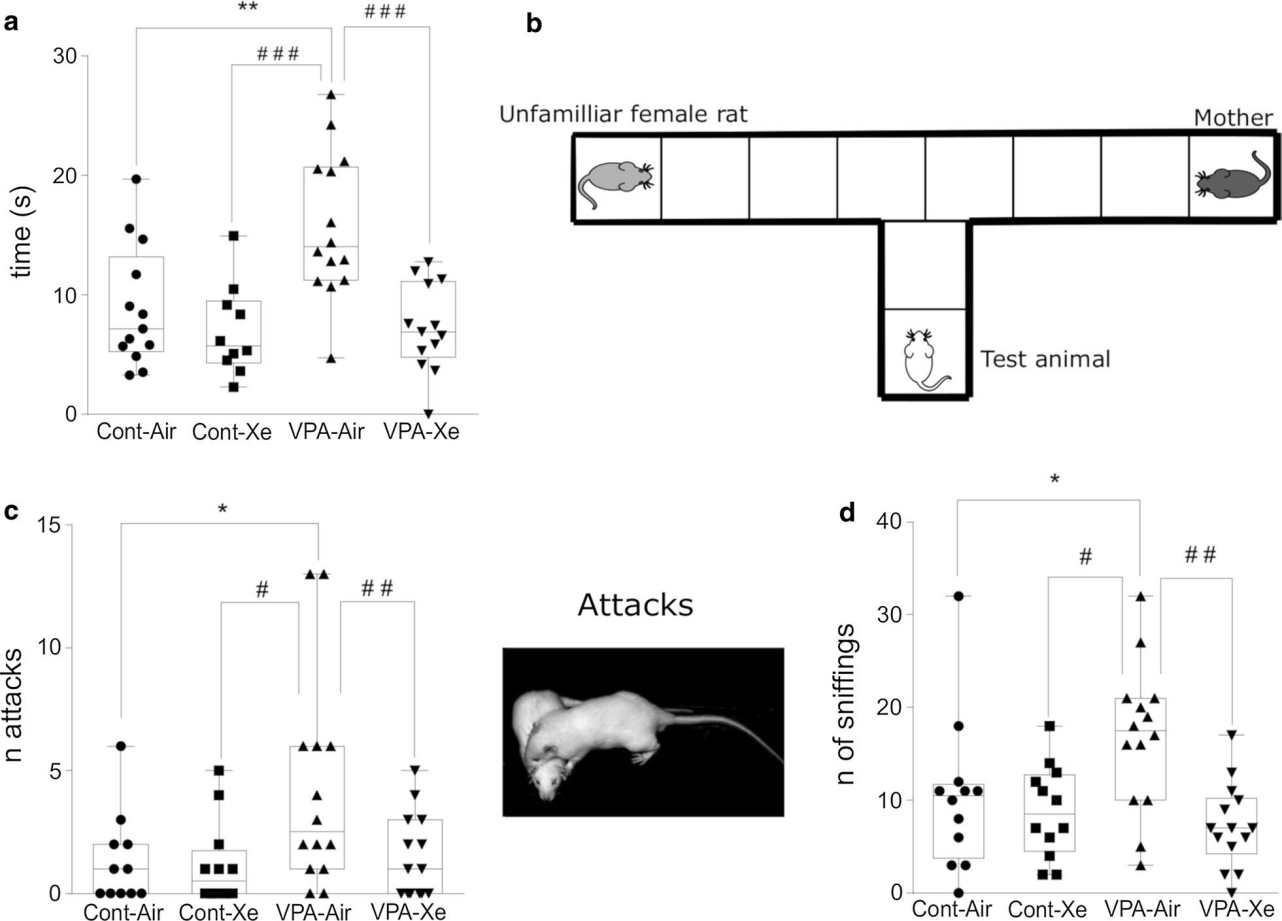
Effects of VPA treatment and acute Xe inhalation on the social behavior of rats. Values represent latency (in seconds) to leave the starting area of the maze (**a**) in the test for social novelty on pnd 28, **b** Schematic representation of apparatus for social novelty test, **c** number of attacks, **d** number of sniffings in the test for social play behavior on pnd 32. *p < 0.05, **p < 0.01 represent significant differences vs. Control group; ^#^p < 0.05, ^##^p < 0.01, ^###^p < 0.001—vs. VPA group (Dunnett’s multiple comparisons test). Data are presented as box and whiskers plot. N = 11–14 rats/treatment

The results of one-way ANOVA revealed a significant effect of treatment (F_3, 48_ = 9.473; p < 0.001) on the latency to leave the starting area, with “VPA” group having larger latency in comparison with “Cont” (p = 0.002) and “Xe” (p < 0.001) group of animals. An acute inhalation of Xe resulted in decrease in the time to leave the starting area in VPA-exposed animals in comparison with VPA animals which received atmospheric air inhalation before the test (p < 0.001), wherein “VPA + Xe” group didn’t differ from “Cont” group (p > 0.99) (Fig. 9a). Xe administration didn’t affect the behavior of healthy animals (p > 0.70 vs. “Cont” group). There was no significant effect of treatment on the time spent with dam (F_3, 48_ = 0.326; p > 0.80) and with unfamiliar adult female (F_3, 48_ = 0.825; p > 0.40).

##### Social play behavior

The results of one-way ANOVA revealed a significant effect of treatment on the number of pinning and pouncing (F_3, 48_ = 3.868; p = 0.015) and the number of sniffings (F_3, 48_ = 5.433; p = 0.003). *Post*-*hoc* analysis revealed an increased number of attacks in “VPA” group in comparison with “Cont” (p = 0.031) and “Xe” (p = 0.016) groups (Fig. 9c). Also, “VPA” group had an increased number of sniffings in comparison with “Cont” (p = 0.049) and “Xe” (p = 0.011) groups (Fig. 9d). The number of attacks were decreased in “VPA + Xe” group in comparison with “VPA” group and didn’t differ in comparison with “Cont” group of rats. In addition, Xe treatment of VPA-exposed animals resulted in decreased number of sniffings (p > 0.40 vs. “Cont”; p = 0.001 vs. “VPA”). Xe exposure didn’t affect the number of attacks and sniffings in healthy rats (p > 0.05 vs. control animals).

#### Forced swim test

**Fig. 10:**
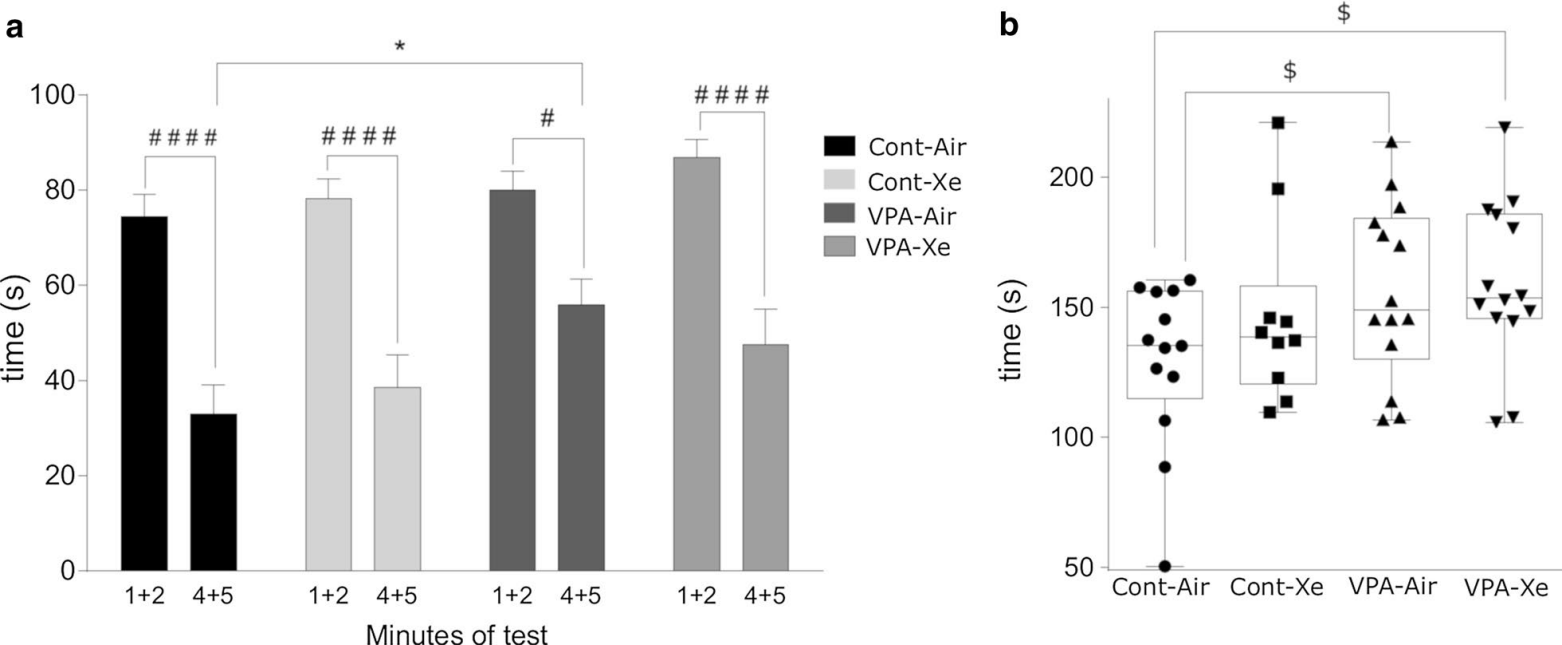
Performance of rats in forced swim test on pnd 35. Values represent climbing activity (in seconds) during the first 2 and last 2 min of observation (**a**) and total climbing time within 5-min observation (**b**). *p  <  0.05 represent significant differences and p ≥ 0.05 represent trend towards differences vs. Control group; ^##^p < 0.05, ^###^p < 0.001, ^####^p < 0.0001—vs. VPA group (Dunnett’s multiple comparisons test). $—0.10 ≥ p ≥ 0.05 represent trend towards differences vs. Control group (Sidak’s multiple comparisons test). Data are presented as box and whiskers plot. N = 11–14 rats/treatment

The result of two-way repeated measures ANOVA revealed a significant effect of treatment (F_3, 44_ = 3.359; p = 0.027) and time (F_1, 44_ = 79.97; p < 0.0001) and no effect on treatment X time interaction (F_3, 44_ = 1.016; p = 0.39) on climbing activity in the FS test. In all experimental groups the duration of climbing behavior was reduced in the second half of the testing (p < 0.02). Post hoc analysis revealed a tendency towards increased climbing in “VPA” (p = 0.096) and “VPA + Xe” (p = 0.053) groups in comparison with “Cont” group of animals (Fig. 10). Moreover, a significant increase in the duration of climbing was revealed in “VPA” group in comparison with “Cont” group during the last 2 min of experiment (p = 0.011). There was no effect of Xe inhalation on climbing behavior in healthy animals (Fig. 10). One-way ANOVA has not revealed any effect of treatment on the time spent immobile (F_3, 44_ = 0.886; p = 0.45) and the number of dives (F_3, 44_ = 1.143; p = 0.34).

## Discussion

### Series 1

In series 1 we assessed the effects of acute subanesthetic doses of Xe on normal rats in a battery of behavioral tests. Of note, this is the first study in which a short-term exposure to Xe gas was used to evaluate such effects. In all of the previous studies animals were treated with 25–70% Xe for at least 20 min and up to 5 h [22, 24, 38–42]. Importantly, pharmacokinetic analysis of exposure to 50% Xe has shown that the maximal concentration of Xe in the rats’ brain is reached within the 1st min of exposure [43]. These data suggest that even a short-term exposure to Xe can affect neurotransmission.

Our results suggest that Xe-exposed rats showed no sign of sedation as there was no decrease in horizontal locomotion and grooming activity in the OF test. Besides a measure of locomotor activity, the OF test measures other factors such as exploratory drive and anxiety-like behavior. Some outcomes, particularly defecation, center time, and activity within the first 5 min, may also measure some aspects of emotionality [44]. The decrease in rears, center entries and time spent in the center of a maze may suggest a suppression of exploratory motivation or enhanced emotionality evoked by novel conditions in adolescent rats after acute inhalation of Xe.

Also, in the EPM test we have observed a trend towards decline in the time spent on the distal edges of the open arms and in the number of head-dips in Xe-treated animals. There were no changes in anxiety-related behaviors between “Control” and “Xe” groups. These results suggest a modulatory effect of Xe inhalation on proclivity of risk-taking behavior and no effect on anxiety level of adolescent rats.

In the FS test we observed a tendency towards decreased time spent immobile and increased latency to first immobilization. The reduction in immobility is usually identified as an antidepressant-like effect [44]. The effects of acute Xe administration resulted in tendency to reduce behavioral despair during forced swimming test.

In addition, an increased latency to leave the closed arm with no changes in other parameters in the OM suggests reduction of exploratory motivation in Xe-exposed rats. The OM was conducted 5 days after the last Xe inhalation, suggesting the prolonged effect of previous Xe exposures. Based on these results we can conclude that 10 min of 25% Xe inhalations is sufficient in modulating behavior of adolescent animals. Wherein, the exposure to Xe didn’t alter either “normal” locomotor activity nor the “normal” anxiety level of rats in the open field, elevated-plus and elevated O-maze tests. At the same time, Xe administration resulted in reduced exploratory motivation and/or emotionality evoked by novel conditions in OF and OM, and slightly decreased the risk-taking behavior in EPM. In addition, Xe has shown ability to reduce behavioral despair of rats in FS. The decreased exploratory motivation in OM suggests delayed, long-term effects of Xe inhalation. There have been no previous studies on behavioral changes in healthy rats after Xe treatment. The behavioral modulatory effects of Xe which was shown in series 1 are probably related to its generalized effect on excitatory/inhibitory balance within the CNS through NMDA inhibition and/or TREK-1 activation [45, 46]. We propose that these effects of Xe might be translated into the treatment of psychoemotional disorders.

### Series 2

The most important finding of this study is demonstration of improved social behavior of VPA-exposed rats after a single inhalation of Xe (25%, 10 min). Prominent inhibitory effect of Xe on NMDA receptors makes this gas an attractive modality for studying pathological conditions involving these receptors. Rats prenatally exposed to VPA show increased NMDAR levels, enhanced NMDAR-dependent LTP, and hyperconnected local neocortical circuits [47, 48]. In the current work, aggressive behavior as well as social anxiety of rats treated with VPA from pnd 6 to pnd 12 at the dose of 150 mg/kg was significantly reduced by acute Xe administration. These data suggest beneficial effect of subanesthetic short-term exposure to Xe and its possible implication in the treatment of psychoemotional disorders such as ASD.

To assess the outcomes of postnatal VPA exposure and the subsequent Xe inhalations in early life we have conducted tests for sensorimotor development of rats. The negative geotaxis test in neonatal rodents is a measure of sensorimotor function, strength and stamina [49]. The gait test is used to assess integrity of the cerebellum and of the muscle tone [50]. Such complex behaviors involve both excitatory and inhibitory pathways which can be affected by endogenous and exogenous substances. Xe inhalation led to an improved integrative sensorimotor response in the negative geotaxis test, but not in gait reflex in healthy animals. It must be noted that raised quadruped posture and locomotion is fully developed in rats by day 16 [35]. It is plausible that Xe affects the development of sensorimotor function but has no effect when it’s fully developed. The possible mechanism of action of Xe may be in modulation of signal transduction through regulation of NMDA receptors. There was no significant effect of postnatal VPA treatment on sensorimotor development of rat pups and no effects of Xe administration in valproate animals. There is a paucity of information on developmental and behavioral outcomes in postnatal VPA models. It has been previously reported that VPA exposure on embryonic day 12.5 didn’t alter the performance of rats in negative geotaxis test [51]. Also, it was shown that exposure to VPA from pnd 6 to pnd 12 at the dose of 150 mg/kg didn’t affect physical development of rats but led to the disruption of motor skills involved in object manipulation [52]. Other studies using prenatal or postnatal VPA exposure have reported its negative effects on sensorimotor development of rodents, but not in all paradigms [51, 53–55].

In series 1 we observed a decrease in exploratory motivation and/or emotionality of rats after exposure to Xe. In series 2 we have observed an effect of Xe administration similar to that in the previous experiment, showing a decrease in locomotion during the first 2 min of the experiment in Xe-exposed healthy and Xe-exposed VPA animals as well as a trend towards a decrease in rearing in “Xe” group. The performance in OF test during the 1st min of experiment measures a reaction to novelty rather that general activity [44]. In support of “low exploratory motivation” theory it should be noted that previous studies reported no sedating effects of xenon after 30 min treatment with 50% Xe/50% O_2_ gas mixture [41]. In our study the exposure to valproate from pnd 6 to pnd 12 didn’t affect animal performance in the OF test. The data on motor activity of rodents in VPA models of ASD’ are quite conflicting. Both increased [53, 56, 57] and unchanged [54, 58] locomotion in valproate-treated animals independent of dose and prenatal/postnatal administration of VPA have been reported. A recent study reported a 2% frequency of co-occurrence of attention-deficit hyperactivity disorder in children with ASD [59], making this comorbid diagnosis rather uncommon in ASD.

The effects of postnatal VPA administration and Xe exposure on exploratory behavior and anxiety were studied in the EPM. Anxiety is the most common comorbid psychiatric symptom in children with ASD, with prevalence rate reaching 40% [60]. Anxious behaviors are also reported in the studies using VPA model of ASD in EPM [53, 55, 58, 61–63]. We should note that in our study “VPA” group of animals has shown a tendency towards anxious behaviors, whereas “VPA + Xe” group has had a pronounced anxiety-like behavior in elevated plus maze. After acute inhalation of Xe healthy animals didn’t differ from controls. Furthermore, in series 1, Xe has shown potency to reduce risk-taking behavior in adolescent rats, but in series 2 these differences were lost probably due to the reduction of the sample size. Thus, it remains unclear whether Xe inhalation enhanced anxiety-like behavior in VPA-exposed rats. Moreover, some authors propose potential effectiveness of Xe inhalations in treatment of anxiety disorders [32, 64]. Still, further studies broadening an understanding of anxiety-like behavior after Xe inhalation in VPA model of ASD’ may be warranted.

The most prominent feature of autism is social impairment. Most of the studies of behavior in VPA model revealed some, but not all impairments of social interaction [55, 58, 65]. In our study we have shown that postnatal exposure to VPA results in increased aggression towards unknown age-matched animals which was expressed as the increase in the number of pinning, pouncing and sniffing. It is plausible that such reaction is associated with negative emotions towards a stranger. In the study of Kataoka et al. [66], female but not male mice prenatally exposed to VPA spent more time sniffing age-matched animal, without affecting allogrooming or aggression. Aggressive behavior is frequent yet poorly understood in children with ASD [67]. Also, we have observed an increase in latency to leave starting area in social novelty test. We suggest that such reaction was caused not only by the new conditions, but also by the acoustic and olfactory signaling from dam and unknown female, which resulted in the so-called “social anxiety” in adolescent rats. It has been estimated that 40% of ASD patients have at least one anxiety disorder, with social anxiety being one of the most common among them (17%) [60]. Xe administration prior to testing resulted in normalization of aggressive behavior as well as anxiety in VPA-exposed rats. There was no change in social behavior in healthy animals after Xe inhalation. It is plausible that Xe’s modulating effect on excitation/inhibition in CNS occurs mainly through its inhibitory effect on excitatory neurotransmission per se.

In the current study we used the forced swim test as a behavioral paradigm that has been found useful in assessing possible antidepressant activity of drugs [44]. In series 1 we have observed a tendency towards antidepressant effects of Xe, which was not replicated in series 2, probably due to a smaller sample size. Though, we have shown a trend towards increase in climbing activity in both VPA-exposed groups, with significant rise of struggling behavior in the second half of the testing in VPA group in comparison with Control group. During testing, animals of all experimental groups have shown a reduction in climbing activity over time. Because struggling behavior consumes a lot of energy, animals tend to employ energy conserving behavior during testing, such as active swimming and passive floating as a successful coping strategy [68]. The increased climbing in the second half of the experiment may indicate an impaired inhibitory reaction (habituation) which is normally formed during the long testing session. This may also indicate an impaired decision-making in VPA animals with increased probability of earlier exhaustion. Pre-exposure to Xe didn’t affect the overall climbing activity but reduced such behavior to control levels in the second half of the experiment. It is plausible that reduced climbing is a beneficial effect as it strategically helps to conserve energy in a stressful environment. There is paucity of information on behavioral changes in FS test in VPA models of autism with one previous study showing increased depressive tendencies in rats which were prenatally exposed to VPA [55].

Valproic acid model may replicate some but not all symptoms of ASD. The most prominent feature of autism is social impairment. In our study VPA treatment from pnd 6 to pnd 12 at a dose of 150 mg/kg led to increased aggression and anxiety. These effects are the most commonly observed signs of autism in clinical practice. We observed neither any physical or sensorimotor impairment in animals in early infancy, nor locomotor disturbances or depressive-like behavior in adolescence. There is evidence that behavioral and developmental outcomes in VPA model of ASD depend on dose and timing of exposure to valproate. A time frame from pnd 6 to pnd 12 reflects sensitive period of functional development of the rats’ brain during which the processes of proliferation, synaptogenesis and myelination are ongoing. Moreover, at pnd 6–8 a peak in the expression level of NMDA receptors occurs in rodents’ brain and this increase is presumably necessary for the developing brain because activation of glutamate system plays a substantial role in the morphogenesis and development of CNS plasticity [69]. Thus, postnatal exposure to VPA may lead to a delayed long-lasting effect mimicking ASD [33].

## Conclusions

This study addresses an important yet under investigated effect of Xe administration on behavioral outcomes in healthy intact animals and in VPA-induced rodent model of autism.

We have shown that acute treatment with subanesthetic dose of Xe in healthy animals leads to:

- improved sensorimotor integration in the negative geotaxis test;
- acute and delayed decrease of exploratory motivation;
- partial decrease in risk-taking and depressive-like behavior.

We have also found that acute inhalations of xenon in VPA-exposed animals led to:

- improvement in social aggressive behavior and anxiety;
- decrease in exploratory motivation;
- normalization of behavior in forced-swim test.

Behavioral modulatory effects of Xe are probably related to its generalized action on excitatory/inhibitory balance within the CNS. Our data suggest that subanesthetic short-term exposures to Xe have beneficial effect on several behavioral modalities and should be further studied in patients with this devastating disease.

## Data Availability

The datasets used and/or analysed during the current study are available from the corresponding author on reasonable request.

## Abbreviations

Xe: xenon
NMDA: *N*-methyl-d-aspartate
ASD: Autism Spectrum Disorder
VPA: valproic acid
PD: panic disorder
OF: open field test
EPM: elevated plus maze test
FS: forced swim test
OM: O-maze test
